# A Systematic Review of Yoga and Meditation for Attention-Deficit/Hyperactivity Disorder in Children

**DOI:** 10.7759/cureus.36143

**Published:** 2023-03-14

**Authors:** Natalie A Gonzalez, Navya Sakhamuri, Sreekartthik Athiyaman, Bhawna Randhi, Sai Dheeraj Gutlapalli, Jingxiong Pu, Maheen F Zaidi, Maithily Patel, Lakshmi Malvika Atluri, Ana P Arcia Franchini

**Affiliations:** 1 Pediatrics, California Institute of Behavioral Neurosciences & Psychology, Fairfield, USA; 2 Internal Medicine, California Institute of Behavioral Neurosciences & Psychology, Fairfield, USA; 3 Medicine, California Institute of Behavioral Neurosciences & Psychology, Fairfield, USA; 4 Psychiatry and Behavioral Sciences, California Institute of Behavioral Neurosciences & Psychology, Fairfield, USA; 5 Research, California Institute of Behavioral Neurosciences & Psychology, Fairfield, USA; 6 Family Medicine, California Institute of Behavioral Neurosciences & Psychology, Fairfield, USA; 7 General Surgery, California Institute of Behavioral Neurosciences & Psychology, Fairfield, USA

**Keywords:** adhd children mindfulness, adhd children yoga, adhd children meditation, meditation therapy, yoga therapy

## Abstract

With attention-deficit/hyperactivity disorder (ADHD) being a prevalent disorder in childhood, it is essential to acknowledge the different adverse effects the disorder can have on the quality of life in children. Therefore, this systematic review focuses mainly on children. Medical therapy, especially stimulants, can have many side effects. Our systematic review aims to evaluate the potential of other non-medical treatment options for ADHD, such as yoga or meditation. We used PubMed and Google Scholar as databases for this systematic review. Using different combinations of medical subheadings (MeSH) and key terms, followed by the application of several inclusion/exclusion criteria and filters to narrow down our search. From an initial 51,675 articles, we selected 10 papers that passed our screening process and quality check to analyze in depth. Yoga and meditation positively affect various symptoms in children with ADHD, including attention, hyperactivity, and impulsive behavior. If done in family group sessions, it also benefited the parents and family dynamics, suggesting a potential option for family therapy. Furthermore, other psychological symptoms, such as anxiety or low self-esteem, appeared to be positively impacted by these interventions. Although yoga and meditation positively influenced children with ADHD, a more in-depth research is necessary with a more significant number of participants and over a more extended period of time. The results of the included studies suggest a substantial benefit. Nonetheless, as the number of studies is limited, at present yoga and meditation could be beneficial as supplemental therapy rather than being used alone as a therapy for ADHD.

## Introduction and background

Attention-deficit/hyperactivity disorder (ADHD) belongs to a group of conditions classified as neurodevelopmental disorders. The prevalence is about 5% in children and an estimated 2.5% in adults. Individuals with this diagnosis struggle to pay attention, experience an urge to be constantly active, and are impulsive. ADHD can also be responsible for cognitive difficulties and executive deficits. Many factors can lead to ADHD, and one significant known factor is genetics. However, the disorder is not yet fully understood [1]. ADHD criteria are described by the Diagnostic and Statistical Manual of Mental Disorders, 5th edition (DSM-5) [2]. To fulfill the criteria for ADHD, symptoms such as inattentiveness, hyperactivity, and impulsivity must occur in two or more settings for a minimum of six months. It must impair an individual’s life socially and academically and be present before 12 years of age. At least six out of 11 symptoms regarding inattentiveness must be present, and a minimum of six out of nine symptoms regarding hyperactivity and impulsiveness to fulfill the criteria for ADHD. If an individual over 17 is diagnosed with ADHD, only five symptoms per category must be present [2]. Various psychiatric comorbidities are prevalent in children with ADHD. Oppositional defiant disorder and obsessive-compulsive disorder, as well as other mental health issues such as anxiety and depression, are commonly associated to the disorder. As expected, children with ADHD can also experience learning difficulties [2].

ADHD has a significant impact on different areas of a child’s life. Children with ADHD struggle to fit in socially, find peers, and form interpersonal relationships. The disorder can also negatively affect the dynamics among family members. It affects the child's academic life, leading to learning difficulties and problems with completing tasks in a timely manner, and procrastination, which further leads to poor academic performance [3].

The cost for individuals diagnosed with attention-deficit/hyperactivity disorder (ADHD) and their families is estimated to range from $143 to $266 billion in the United States [4]. The high prevalence among children (5%) and adults (2.5%) is the reason for this disorder’s enormous economic impact [1]. Other than being a financial burden, its diagnosis can be mentally and physically challenging.

Effective therapy options can sometimes be challenging. Adverse long-term effects can occur if children do not receive early or efficient therapy [1]. These include failing in academic settings, hardship finding employment, and further keeping them due to problems in executing tasks in time or building good interpersonal relationships with colleagues. Due to difficulty in focusing and being easily distractable, individuals with ADHD also have a higher risk of having traffic accidents [3]. Treatment options vary from non-medication options to various pharmaceuticals categorized as stimulants and non-stimulants. Stimulants (methylphenidate or amphetamines) can result in a variety of severe side effects. It can affect sleep, lead to less appetite, and possibly even cause growth problems regarding height and weight. A study linked stimulants to high blood pressure as well as increased heart rate and palpitations and researchers have even proposed that stimulants could increase the risk of suicide [1]. Therefore, evaluating the risks and benefits of pharmacological treatments is extremely important.

Taking non-medical treatments, such as cognitive behavioral therapy (CBT), into account can be a beneficial way to support children with ADHD [2]. The focus of CBT is to positively highlight the desired behavior. Depending on the age, the therapy is either focused on the person affected (adolescents) or their primary caregivers (young children). Different forms of possible therapy include training in socializing, self-management, and organization. Not only does it positively influence children regarding their behavior, but also the parents and their relationship with their child [2]. Another alternative or additional therapy that could be promising in the matter of improving symptoms of ADHD is the use of yoga and meditation [2]. Yoga is a practice based on Indian traditions and philosophy that has been used for centuries to improve mental and physical health. Yoga aims to unite the mind and body and positively influence concentration and focus. Practicing yoga also showed to alleviate stress in sick patients [5]. Yoga is linked to better health and improvement in psychiatric disorders such as depression and anxiety [5]. The purpose of meditation is to create a connection between the mind and the body. It helps to achieve a feeling of balance and calmness and is known to reduce stress levels. Additionally, it improves self-control and the capacity to bring attention back to something soothing [6]. 

To highlight the importance of this topic, we want to briefly mention ongoing research on ADHD, meditation, and yoga. The National Institutes of Health database for clinical trials reports an ongoing clinical trial, “Mindfulness-Based ADHD Treatment for Children: A Feasibility Study (MBAT-C).” It is a randomized controlled trial that includes 45 children between the ages of seven and 13 with ADHD. The plan is to complete the study by June 2023. The main focus is to assess the effectiveness of mindfulness-based ADHD treatment for children (MBAT-C) by evaluating ADHD symptoms before and after treatment [7].

As there is a relatively small number of clinical trials on meditation and yoga as therapy options for ADHD, we decided to conduct a systematic review to look further into possible therapeutical benefits for ADHD patients [2]. With children being a vulnerable group in our society, our goal is to evaluate if non-medical treatments such as yoga or meditation could add to a higher quality of life, reduce ADHD symptoms, and possibly even replace medications such as stimulants.

## Review

Methods 

**Figure 1:**
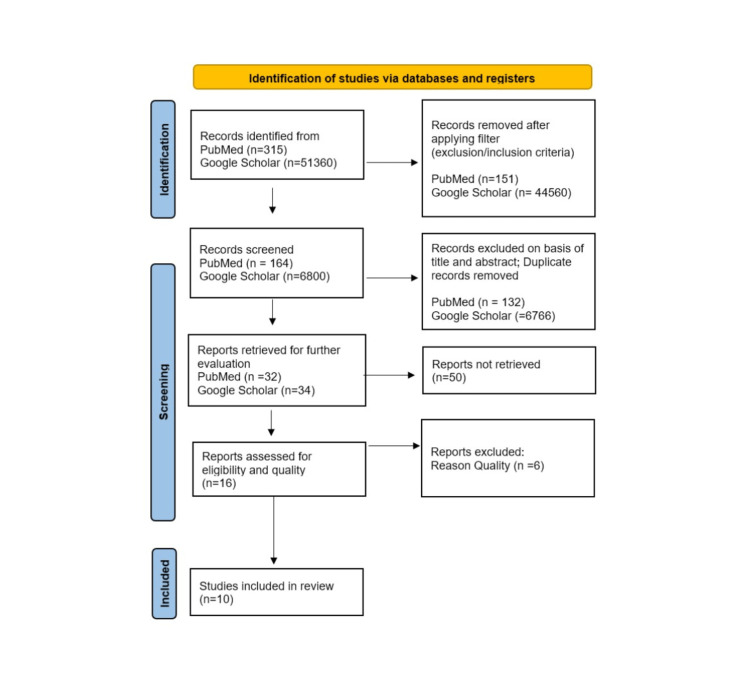
PRISMA flowchart of selected studies. PRISMA: preferred reporting items for systematic reviews and meta-analyses.

This systematic review followed the preferred reporting items for systematic reviews and meta-analyses (PRISMA) guidelines 2020 [8]. The PRISMA flowchart in Figure 1 shows the process of identification and elimination of the publications used [8].

Search Strategy

The two primary databases used were PubMed and Google Scholar. Initially, we searched all key terms individually and came up with a very high number of studies. Therefore, we decided to combine our key terms to come up with more specific results. The key terms "ADHD," "children," "yoga," "meditation," "mindfulness," "therapy," and "treatment" were used in different combinations for each database. For PubMed, we used the following key term combinations: “ADHD children meditation,” “ADHD children yoga,” “ADHD children mindfulness,” “ADHD treatment yoga mindfulness,” “ADHD treatment meditation mindfulness,” “ADHD yoga,” and “ADHD meditation.” For Google Scholar, we used “ADHD children meditation therapy,” “ADHD children yoga therapy,” “ADHD children yoga mindfulness,” and “ADHD children meditation mindfulness” as search terms. In PubMed's advanced search, we used the medical subheadings (MeSH) yoga, meditation, and ADHD in different combinations to search for articles: ADHD AND Yoga AND Meditation, ADHD AND Yoga, and ADHD AND Meditation. We then applied our filters and exclusion/inclusion criteria to the total number of articles found, excluding those that did not meet our specifications. We further screened the remaining articles based on title and abstract and removed duplicates. After further evaluation, we did not retrieve 50 reports based on their content not adding value to our topic. We evaluated the remaining articles and did a quality assessment of those we retrieved. The final number of articles included in our search that met our criteria is 10.

Inclusion Criteria

We included only articles in the English language. Further, we only selected papers from 2002 until the day of our data search on June 10, 2022. We applied the following filter for the population: children ages zero to 18 years old.

Exclusion Criteria

We excluded every article not written in the English language from our data collection. Also, we removed all published articles before 2002 from our PubMed search and excluded all articles that focused on ages other than zero to 18 years old. We did not select articles that were not free for further evaluation.

Results

The number of articles we found with our search strategy was 315 in PubMed and 51,360 in Google Scholar. After applying our filters and exclusion/inclusion criteria, we excluded 151 articles in PubMed and 44,560 articles in Google Scholar. We further screened the remaining 164 articles in PubMed and 6800 articles in Google Scholar and were able to delete 132 articles in PubMed and 6766 articles in Google Scholar based on title and abstract; we also removed all duplicates. We evaluated the remaining 32 articles in PubMed and 34 articles in Google Scholar. After not retrieving 50 articles, we did a quality assessment on 16 articles and excluded six articles for not meeting our criteria. The final number of articles included in our systematic review is 10. Out of the 10 eligible articles, there was one systematic review, one meta-analysis, one review article, three randomized controlled trials, two clinical trials, and two qualitative studies. For a more detailed description of our selected papers, see Table 1.

**Table 1:** Important studies exploring ADHD and yoga as a therapy option for ADHD in children. MBT: mind-body therapies; ADHD: attention-deficit/hyperactivity disorder; USA: United States of America; RCT: randomized controlled trial; T1: time one; KiTAP: kinder test of attentional performance; HRV: heart rate variability; MOM: mindfulness-oriented meditation program; MBI: mindfulness-based intervention; CAU: care-as-usual; UK: United Kingdom.

Study	Year of publication	Location	Type of study	Total patient population	Outcome
Barranco-Ruiz et al. [9]	2019	Spain	Systematic review	Three hundred eighty-eight children age 5-18 years	Eleven out of 12 studies suggested a positive result between MBT and symptoms of ADHD.
Bigelow et al. [10]	2021	Canada	Qualitative study	Sixteen children age 10-14 years	One meditation session improved executive functions. Meditation influenced inhibitory control positively. Active exercise increases the mood and overall well-being of children.
Cohen et al. [11]	2018	USA	RCT	Twenty-three children age 3-5 years	Group one (yoga first) showed improvement in inattention. After T1 (six weeks), group one reacted faster on the KiTAP go/no-go task. The distractibility task had fewer omission mistakes but more commission mistakes than the control group. None of the groups showed a difference in HRV.
Krisanaprakornkit et al. [12]	2010	Thailand	Review	Eighty-three participants age 6-13 years	Studies have shown no appreciable difference between the meditation therapy group and the group that received drug therapy with ADHD on the teacher rating ADHD scale.
Santonastaso et al. [13]	2020	Italy	Clinical trial	Twenty-five children age 7-11 years	MOM program showed improving ADHD symptoms in participants compared to the control group.
Saxena et al. [14]	2020	USA	Clinical trial	One hundred seventy-four children age 14-15 years	After 12 weeks of either being in the yoga group or the control group, the self-questionnaire of the children showed improvement regarding inattention and hyperactivity in the yoga group. Neither group showed changes in stress levels.
Siebelink et al. [15]	2021	Netherlands	Qualitative study	Sixty-nine families with children age 9-16 years	Interviews after participating for eight weeks in a group-based MBI training named “MYmind” indicated positive effects on the symptoms of ADHD.
Siebelink et al. [16]	2022	Netherlands	RCT	One hundred and three children age 8-16 years and their parents	The combination of MBI and CAU did not have a significant difference compared to CAU. However, some children experience some improvement. The therapies had more effects on parents and also lasted for a longer time.
Thomas et al. [17]	2021	UK	RCT	Seventy-nine participants age 23-27 years	The group who received modafinil showed better attention and a higher state of mindfulness, but it did not influence mind-wandering. The meditation experience in general was not enhanced compared to the group who received the placebo drug.
Xue et al. [18]	2019	China	Meta-analysis	Six hundred eighty-two participants (210 children age 5-8 years, 472 adults 18-65 years)	Results showed that MBI training positively affects ADHD symptoms, such as impulsive behavior, attention span, and hyperactivity.

Quality Check

After selecting the final articles, they underwent a quality appraisal tool. For all systematic reviews and meta-analyses, we used the assessment of multiple systematic reviews (AMSTAR) criteria. For reviews or other papers we were not able to further classify, we used the scale for the assessment of narrative review articles (SANRAs) scale for quality assessment. For all randomized controlled trials, the Cochrane risk of bias tool was used to assess quality. For other clinical trials, we used the Newcastle-Ottawa Tool Scale. Finally, we used the Critical Appraisal Skills Programme (CASP) checklist for qualitative studies.

Discussion

ADHD and Yoga Therapy

A randomized controlled trial by Cohen et al. evaluated the effect of yoga therapy on 23 children aged three to five years [11]. The study used a parent and teacher ADHD Rating Scale-IV (ADHD RS-IV) Preschool Version and Strengths and Difficulties Questionnaire (SDQ) to evaluate the effect of the intervention. The Kinder Test of Attentional Performance (KiTAP) objectively evaluated attention. The heart rate variability (HRV) assessed the degree of self-regulation in the participants. Children participated in yoga therapy for over six weeks in both a school and home setting. First, group one (n=12) started with yoga from week one to six, followed by group two (n=11), which did yoga from week six to twelve. Possible effects of yoga therapy were evaluated in the beginning, after six weeks as time one (T1), after twelve weeks as time six (T6), and finally three months later as time two (T2) [11]. Group one and group two showed no differences after the first assessment at baseline. Group one at T1 improved better on the KiTAP go/no-go task. On the one hand, their reaction was faster, and they made fewer omission mistakes; on the other hand, they made more commission mistakes than in group two. The study also showed that if children in group one expressed more symptoms from the beginning, they had significantly better improvements after the yoga therapy than children with fewer symptoms. They also reported at the follow-up rating that the improvement lasted. During the study, the measures of the HRV did not change in either group [11]. The study concluded that using yoga as therapy improved ADHD symptoms, especially in children that experienced more intense symptoms at baseline, but not so much for children with fewer symptoms. The recommendation is that additional studies with more participants and an active control group are necessary to better assess yoga’s effect on symptoms in children with ADHD [11]. Saxena et al. conducted a trial in 2020 that included 174 children 14 to 15 years old [14]. The purpose was to evaluate the effect of Hatha yoga on ADHD symptoms such as inattention, hyperactivity, and distress. The study assigned 123 students to a yoga group and 51 to a control group for 12 weeks. The children in the yoga group participated in yoga classes two times per week. Each Hatha yoga class ended with a short meditation session [14]. The students completed a self-questionnaire regarding their experience at baseline and after 12 weeks of intervention. The yoga group showed improved attention, whereas the control group did not. The children participating in yoga also showed improvements in hyperactivity compared to the control group. The stress levels stayed the same in either group. Therefore, yoga sessions seem to influence ADHD symptoms positively [14]. However, a few limitations apply to this study. First of all, the self-questionnaire of the students evaluates the symptoms as subjective rather than objective. The study also suggests that each yoga session consisting of only 25 minutes may have been too short. Therefore, further research with more objective measures and more frequent or prolonged yoga sessions is necessary [14].

ADHD and Meditation Therapy

A review from Krisanaprakornkit et al. investigated the use of meditation therapy for ADHD. The review selected a total of four randomized controlled trials with 83 participants. Only two studies' data were sufficient for the review's meta-analysis [12]. The study conducted the intervention over four weeks, with different groups receiving either meditation, drugs, or standard treatment with no drugs as therapy. On the teacher rating ADHD scale, researchers did not find an appreciable difference between the group that received meditation as an intervention and the group that received drug therapy regarding ADHD symptoms. Further, comparing the meditation therapy group with the standard therapy group with no medications after four weeks did not show significant changes on the teacher rating ADHD scale or the distraction test [12]. Another study assigned the groups to either receive meditation, relaxation training, or controls with drug therapy. The group that received meditation or relaxation as therapy showed reduced impulsivity, and parents reported a better attitude at home. The meditation group showed enhanced attention. The study did not report behavior improvements at school in either group [12]. The limitation of this review is the low number of studies and participants included, showing the need for further studies in this matter. Therefore, the study cannot report the results regarding the beneficial use of meditation as therapy for ADHD with certainty [12].

A qualitative study from Bigelow et al. studied the effects of mindfulness meditation and exercise on executive functions and emotional well-being in children diagnosed with ADHD [10]. Included were 16 children between 10 and 14 years. The study evaluated various aspects of executive function, such as inhibitory self-control, short-term memory, shifting attention between tasks, and well-being, such as emotions and self-efficacy. Researchers measured the symptoms in participants before and after each intervention. The interventions used were exercising for 10 minutes, mindfulness meditation for 10 minutes, and 10 minutes of reading as a control. Parents were instructed not to give ADHD drugs to their children before the days of the interventions [10]. The study used a free smartphone application named Smiling Mind for all meditation sessions. The results showed that already one mediation session enhanced executive function compared to the other interventions. Meditation also influenced inhibitory control positively. Self-efficacy and overall well-being did not seem to be affected by the intervention. However, acute exercise positively enhanced them. Otherwise, children did not show any effects on executive function after exercise [10]. In conclusion, this study showed that meditation positively impacts executive functions, whereas exercise was a better way to improve psycho-emotional well-being [10].

Siebelink et al. conducted a qualitative study on a group-based mindfulness-based intervention (MBI) training named Mymind [15]. The primary purpose of this study was to investigate factors that either encouraged or discouraged children and their parents from being part of MBI training, as well as the effects on them. Sixty-nine families participated in a mindfulness-based intervention (MBI) training over eight weeks. Group sessions were conducted simultaneously and separately for the children with ADHD and their parents for the most part, with only a few sessions together. For interviews afterward, researchers chose a sample of 17 children between nine and 16 years with ADHD, 20 parents, and three mindfulness teachers who led the program [15]. The MBI training included different meditation practices such as sitting meditation, body scan, breathing space, and some yoga exercises in between. The interviews showed different perceptions of possible barriers or facilitators for the MBI training [15]. An obstacle for both children and parents was that the MBI training was very time-consuming. Many parents felt that they would have needed more sessions for them to integrate it into their everyday lives. Children sometimes reported meditation was as challenging as they had the urge to move. However, the additionally integrated yoga exercises helped them in that matter. For the mindfulness teachers, the limitation was to finish the MBI sessions and the protocol on time. They would prefer to work more independently, especially if parents wanted to open up and share their stories [15]. The facilitators for both children and family were that they could share their experience in this training and support each other. Especially the training sessions together gave them a sense of being more connected with each other. They all appreciated the different aspects of the training. In particular, the “breathing space” was named to be successfully integrated into their lives. One of the most important facilitators for parents was the relief of expressing their feelings and talking about their experience with other parents who also have children with ADHD [15]. The study concludes that the MBI training program Mymind shows some positive effects regarding ADHD symptoms. Meditation practices helped to raise awareness within themselves and not be so harsh on themselves. Children and parents felt more sense of calmness and better control of their emotions [15].

Further, Siebelink et al. also conducted a randomized controlled trial for children with ADHD and their parents named Mindfulness Training for Children with ADHD and Mindful Parenting (MindChamp) [16]. The study compared the combination of mindfulness-based intervention (MBI) training and care-as-usual (MBI+CAU) with CAU-only. The study included 103 children between eight and 16 years who still had symptoms of ADHD after care-as-usual and their parents. Care-as-usual allowed children to receive therapy but MBI outside the study for their ADHD. About 81% of children reported taking ADHD drugs at the beginning of the study. Researchers measured the effects at the beginning as time zero (T0), after the treatment as time one (T1), and follow-up after two as time two (T2) and six months as time three (T3). Participants followed the meditation sessions over eight weeks and repeated them after eight weeks as a refresher [16]. The combination of MBI and CAU did not show a significant benefit regarding self-control in children. However, MBI still showed positive effects on both children and parents [16]. Parents felt more mindful when caring for their children and were kinder to themselves. Children showed significant improvements in attention, better control of their impulses, and hyperactivity. Additionally, the MBI group showed positive effects in various mental health aspects, such as better sleep and being less fearful and introverted. The outcome suggests that MBI in a family can be a beneficial additional therapy option for some children and their parents [16].

A clinical trial from Santonastaso et al. compared the effect of mindfulness-oriented meditation (MOM) to an Emotion Education Program (EEP) as a control [13]. Included were 25 children between seven and 11 years, from where 15 children were part of the MOM training and 10 children were in the control EEP group. The trial lasted over eight weeks, with three training sessions per week [13]. The meditation sessions consisted of being mindful of the breath, body, feelings, and thoughts. The control group first listened to and then analyzed the book “Six Pixies in My Heart,” which is about a sensitive boy who does not want to face his emotions so no one can accuse him of being too emotional. However, finally, he accepts that it is crucial to experience the good and the harmful spectrums of feelings and comes to peace with being sensitive. As a result, the children received education about their emotions in various situations and how to be more attentive to them throughout the day [13]. Children who participated in the MOM training showed a better improvement in regulating their attention and emotions compared to the EEP control group. The results suggest that meditation could affect the behavior and executive functions such as inhibitory self-control, short-term memory, and shifting attention between tasks of children with ADHD positive [13]. As one of the limitations named in this study was the small number of participants, more research is needed to evaluate MOM training in a more significant number of children with ADHD for a longer duration [13]. 

Barranco-Ruiz et al. conducted a systematic review to evaluate the effectiveness of mind-body therapies (MBTs) on children ages five to 18 with ADHD symptoms [9]. Most studies included had one MBT intervention per week with a variation in length between 40 and 150 minutes, were based in a school setting, and were primarily conducted for eight weeks. ADHD symptoms were measured using questionnaires, scales, or visual tests. Three studies focused on using yoga therapy, whereas nine chose mindfulness exercises, such as guided and sitting meditations [9]. Eleven out of 12 studies suggested a reduction of ADHD symptoms in children after MBT-related interventions. Children showed enhanced attention, behavioral self-control, and less hyperactivity. Other psychological symptoms, such as fearfulness, insecurity, and distressing emotions also improved after MBT training [9]. Limitations were the inclusion of many low-quality studies and a small number of participants. More research in this field is necessary, focusing on a higher number of children [9].

Xue et al. conducted a meta-analysis to analyze the impact of mindfulness-based interventions (MBIs) on ADHD [18]. The focus was on the primary symptoms, such as inattentiveness, impulsive behavior, and hyperactivity. Researchers compared 11 studies with a total of 682 participants [18]. Compared to the control group, the studies showed that ADHD symptoms improved with MBI training. Mainly, MBI training influenced inattention more than impulsive behavior or hyperactivity. It also helped participants to regulate their emotions better. In six included studies, participants were followed months after the MBI training, showing positive results that lasted for the following two to eight months [18]. Limitations of the included studies were the small number of participants and the quality of some studies. Besides one analyzed the high-quality study, eight were only moderate, and two were low in grade. The study highlights that more in-depth research is necessary to explore the effect of MBI, especially over a more extended period [18].

Effects of the Combination of Medication and Meditation 

Thomas et al. investigated the effect of the stimulant modafinil on mindfulness and whether the combination of the drug and mindfulness training, such as meditation, can achieve better results [17]. The study mentioned that individuals with ADHD who struggle to focus and pay attention might have trouble gaining positive effects from meditation and, therefore, may achieve better results if they practice mindfulness with a combination of stimulant use. The randomized controlled trial recruited 79 healthy participants. They were assigned randomly to receive either a single dose of a placebo drug or modafinil. The interventions used were relaxation or mindfulness training. The relaxation training contained breathing exercises and muscle relaxation, whereas the mindfulness training used meditation [17]. Researchers assessed the results before and after taking the placebo or drug and after the intervention relaxation or mindfulness training. The study conducted one last follow-up assessment after one week, where participants should continue with relaxation or meditation without drug use [17]. Results showed that the use of modafinil positively affected achieving attention and mindfulness but did not enhance the meditation experience more than the group who received the placebo drug. Also, modafinil did not show to influence mind-wandering. However, more participants who took modafinil continued their relaxation or mindfulness practice for one week compared to participants who took the placebo drug [17]. The study suggests further research, especially in individuals with ADHD, to conclude the combination of modafinil and mindfulness compared to mindfulness [17].

Limitations

Some limitations of our systematic review include only retrieving studies in the English language since 2002. We decided to only select studies of children from birth until 18 years old. Therefore, we do not know if there are other studies conducted on yoga and meditation therapies with adults. As there is a limited number of studies for either yoga or meditation therapy in children with ADHD, many possible effects are still unclear. A further limitation of this study is that we only chose open-access articles for further evaluation.

## Conclusions

Our study evaluated yoga and meditation for children with attention-deficit/hyperactivity disorder (ADHD) and assessed the possibility of these interventions substituting or replacing medical therapy. Our included studies showed that yoga could improve ADHD symptoms and has the potential to be an additional therapy. Further, yoga and meditation therapy positively impacted the relationship dynamics within a family with ADHD, considering it a promising option for family therapy. In particular, meditation also positively affected the parents and helped them feel calmer and more mindful when caring for their children with ADHD. Both interventions positively influenced other aspects of mental health, such as anxiety, insecurity, and stress. Meditation had a positive effect on a variety of ADHD symptoms. For example, executive function, inhibitory control, hyperactivity, impulsivity, attention, and management of emotions. In conclusion, as yoga and meditation positively impact various symptoms of ADHD, they have a significant potential to be a bigger part of the therapy in the future. However, as the number of participants in our included studies was primarily small, we cannot conclude that yoga or meditation therapy could replace medication therapy in total. We suggest that it could be a great way to supplement treatment and support children in achieving a better quality of life. Therefore, it would be essential to do more in-depth research, favorable with a higher number of participants and over a more extended period.
